# Nevoid Basal Cell Carcinoma Syndrome - Clinical and Radiological Findings of Three Cases

**DOI:** 10.7759/cureus.727

**Published:** 2016-08-08

**Authors:** Ibrahim K Ali, Freny R Karjodkar, Kaustubh Sansare, Prashant Salve, Amaresh Dora, Shikha Goyal

**Affiliations:** 1 Oral Medicine and Radiology, Nair Hospital Dental College

**Keywords:** nevoid basal cell carcinoma syndrome, keratocystic odontogenic tumor

## Abstract

Nevoid basal cell carcinoma syndrome (NBCCS) is an autosomal dominant disorder, characterized by skeletal anomalies and multiple keratocystic odontogenic tumors of the jaws. The skeletal anomalies of this syndrome are mandibular prognathism, bossing of frontal and parietal bones, high-arched palate, and bifid rib. We report three cases with NBCCS, emphasizing the clinical and radiographic findings, the importance of the early diagnosis of NBCCS, and a preventive multidisciplinary approach in the management of NBCCS.

## Introduction

Nevoid basal cell carcinoma syndrome (NBCCS), also known as Gorlin-Goltz syndrome, is an uncommon multisystemic disorder, inherited in the autosomal dominant pattern, which shows a high level of penetrance and variable expressiveness. This syndrome is also designated as basal cell nevus syndrome, multiple basal cell carcinoma syndromes, jaw cysts-basal cell tumors-skeletal anomalies syndrome, and bifid rib syndrome. NBCCS commonly results from mutations in the PTCH1 gene located on chromosome 9q22.3-q31 [1]. In 1960, Gorlin and Goltz reported the classical triad of multiple basal cell carcinoma, odontogenic keratocyst, and bifid ribs to establish the diagnosis of NBCCS. In addition to this triad, other characteristic features of NBCCS are palmar and plantar epidermal pits, calcification of the falx cerebri, spine and rib anomalies, macrocephaly, frontal bossing, hypertelorism, medulloblastoma, ovarian fibroma, cleft lip and/or palate, and several other developmental malformations [2-3]. This article reports case series of patients diagnosed with Gorlin-Goltz syndrome, thus, accentuating the need for meticulous clinical and radiographic examination of the affected patients.

## Case presentation

Informed patient consent was obtained from the parents of the children in Cases 1 and 2. In the case of the third patient, informed patient consent was obtained as well as permission to use his photograph in this article.

### Case 1

**Figure 1:**
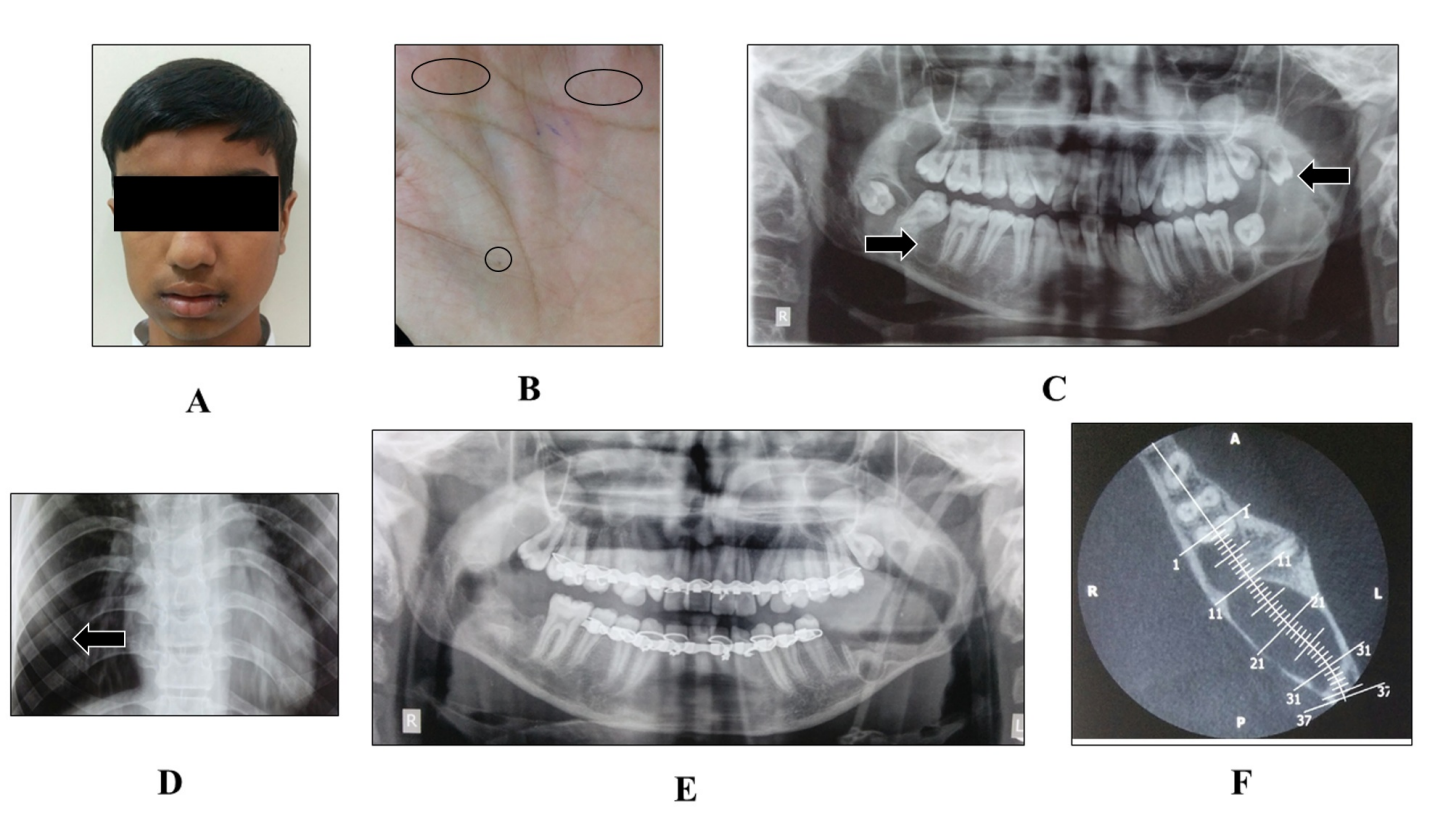
(A) Extraoral profile; (B) General examination showing palmar pits; (C) Panoramic radiograph; (D) Chest radiograph; (F) Cone beam CT scan (axial view); (E) Postoperative panoramic radiograph

A 16-year-old male reported to the Oral Medicine unit with the complaint of pain and swelling in the right and left posterior mandibular region for two weeks' time. The patient’s medical history and family history were noncontributory. His general examination revealed frontal bossing, wide nasal bridge, hypertelorism (Figure 1A), and palmar and plantar pits (Figure 1B). Extraorally, the facial profile was asymmetrical due to swelling in the right side of the face. The temperature of the overlying skin was normal. Intraorally, an irregular, ill-defined swelling expanding the lingual and buccal cortex was present in the right side of the mandible with bony hard consistency. Blood investigations (CBC, Hb, BT, CT, ESR), serum calcium, phosphorus, and alkaline phosphatase were within normal limits. A panoramic radiograph revealed four well-defined radiolucencies suggestive of a benign odontogenic cyst (Figure 1C). A chest radiograph demonstrated bifid ribs (Figure 1D). Cone beam CT scan illustrated the expansion of buccal and lingual cortical plates, perforation of the lingual cortical plate, and a horizontally impacted mandibular permanent left second molar (Figure 1F). NBCCS was established as a provisional diagnosis, based on the clinical and radiological findings. Mandibular molars involved in the lesion were extracted surgically. Multiple mandibular lesions were enucleated, and Carnoy’s solution was applied to peripheral osseous walls. The histopathologic examination of the enucleated tissue demonstrated features suggestive of a keratocystic odontogenic tumor (KCOT). Healing of the enucleated sites was observed on follow-up visits (six months) without any sign of recurrence (Figure 1E).

### Case 2

**Figure 2:**
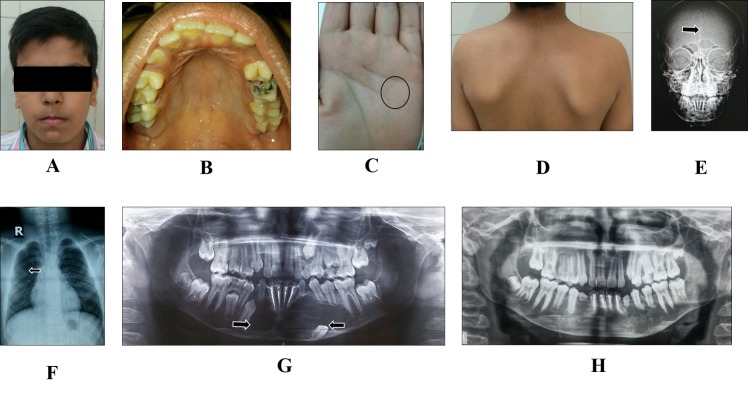
(A) Extraoral profile; (B) Intraoral examination; (C, D) Physical examination; (E) PA mandible radiograph; (F) Chest radiograph; (G) Panoramic radiograph; (H) Postoperative radiograph

A 10-year-old male reported to the Oral Medicine unit with the complaint of pain on the left side of the face. The patient’s medical history and family history were noncontributory. The general examination revealed macrocephaly, ocular hypertelorism (Figure 2A), palmar and plantar pits (Figure 2C), and a Sprengel scapular deformity (Figure 2D). On extraoral examination, facial symmetry was noted. Intraorally, swelling in the mandibular right parasymphyseal region and high arched palate (Figure 2B) was observed. Routine blood investigations (CBC, Hb, BT, CT, and ESR), serum calcium, phosphorus, and alkaline phosphatase were normal. A posterior to anterior (PA) mandible radiograph revealed mild calcification of the falx cerebri (Figure 2E). A chest radiograph demonstrated bifid ribs (Figure 2F). A panoramic radiograph revealed three well-defined radiolucencies suggestive of a benign odontogenic cyst (Figure 2G). NBCCS was established as a provisional diagnosis, based on the clinical and radiological findings. The patient was managed surgically under general anesthesia. Surgical extraction of an inferiorly displaced mandibular permanent left canine was performed. Multiple mandibular lesions were enucleated, and Carnoy’s solution was applied to peripheral osseous walls. The histopathologic examination of the enucleated tissue demonstrated features suggestive of KCOT. Healing of the enucleated sites was observed on follow-up visits (six months) without any sign of recurrence (Figure 2H).

### Case 3

**Figure 3:**
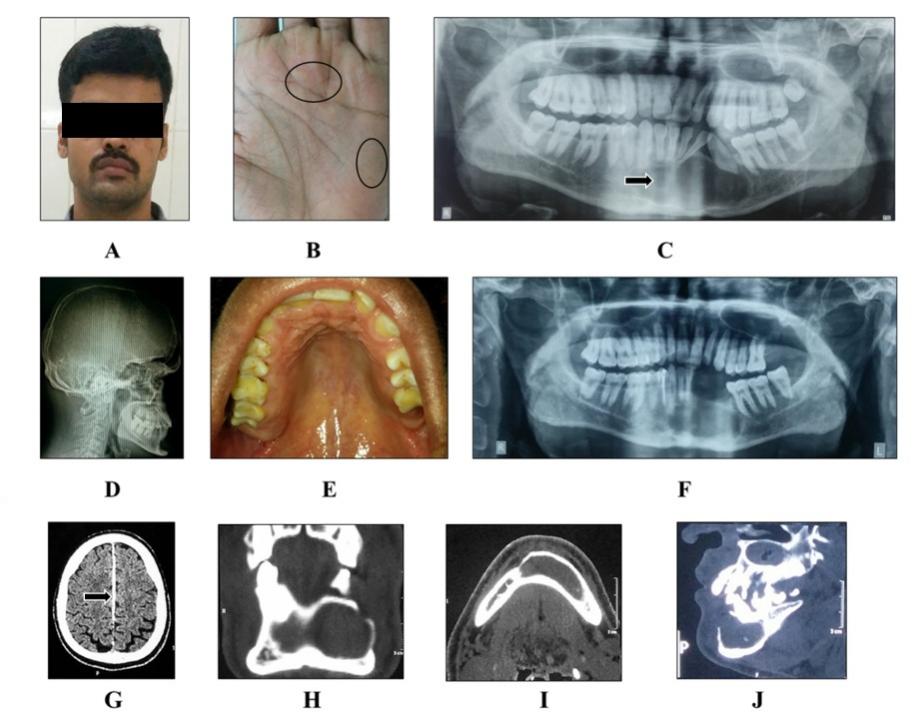
(A) Extraoral profile; (B) Palmar pits; (C) Panoramic radiograph; (D) Lateral cephalogram; (E) Intraoral examination, (F) Postoperative panoramic radiograph; (G, H, I, J) CT scan

A 26-year-old male patient reported to Oral Medicine unit with the complaint of malaligned teeth. The patient’s medical history and family history were noncontributory. On general examination, the presence of macrocephaly, ocular hypertelorism (Figure 3A), and palmar and plantar pits (Figure 3B) were documented. On extraoral examination, facial symmetry was noted. On intraoral examination, displaced crowns of mandibular permanent left lateral incisor and mandibular left canine, along with a high arched palate, were noted (Figure 3E). Routine blood investigations (CBC, Hb, BT, CT, ESR), serum calcium, phosphorus, and alkaline phosphatase were within normal limits. A panoramic radiograph revealed well-defined radiolucency suggestive of a benign odontogenic cyst (Figure 3C). Lateral cephalogram showed bridging of the sella (Figure 3D). A CT scan showed calcification of the falx cerebri, along with the expansion of the buccal and lingual cortical plates and perforation of the buccal cortical plate (Figures 3G-3J). NBCCS was established as a provisional diagnosis, based on the clinical and radiological findings. The patient was managed surgically under general anesthesia. Multiple mandibular lesions were enucleated, and Carnoy’s solution was applied to the peripheral osseous walls. Histopathologic examination of the enucleated tissue demonstrated features suggestive of KCOT. Healing of enucleated sites was observed on follow-up visits (six months) without any signs of recurrence (Figure 3F).

Clinical and radiological findings in this current case series with NBCCS are shown in Table 1.

**Table 1:** Major Criteria, Minor Criteria and Other Clinicoradiographic Findings in Three Cases KCOT = keratocystic odontogenic tumor

Case no.	Major criteria	Minor criteria	Other findings
1	Bifid ribs Palmar and plantar pits Histopathologically diagnosed KCOT	Ocular hypertelorism Frontal bossing Macrocephaly	Wide nasal bridge High-arched palate Malocclusion Over-retained deciduous teeth
2	Bifid ribs Palmar and plantar pits Calcification of falx cerebri Histopathologically diagnosed KCOT	Ocular hypertelorism Frontal bossing Macrocephaly Sprengel deformity	Wide nasal bridge High-arched palate Malocclusion
3	Palmar and plantar pits Calcification of falx cerebri Histopathologically diagnosed KCOT	Ocular hypertelorism Frontal bossing Macrocephaly	Wide nasal bridge High-arched palate Fused eyebrows Malocclusion Impacted maxillary left 3^rd^ molar

## Discussion

In 1993, Evans, et al. [4] first established major and minor criteria for the diagnosis of NBCCS, which was later modified by Kimonis, et al. [5] in 2004. The presence of two major and one minor or one major and three minor criteria are essential to confirm the diagnosis of NBCCS [4-5]. Major criteria include multiple basal cell carcinomas, keratocystic odontogenic tumor of the jaw (histologically proven), bifid or fused ribs, calcification of the falx cerebri, palmar and/or plantar pits (three or more), and a  first-degree relative with a nevoid basal cell carcinoma syndrome. Minor criteria consist of skeletal manifestations such as a pectus deformity, a Sprengel deformity, syndactyly of the digits, bridging of the sella turcica, vertebral anomalies, frontal bossing, hypertelorism, cleft lip or cleft palate, macrocephaly, ovarian fibroma, and medulloblastoma [4-5]. Multiple KCOTs are the most persistent and characteristic symptom of NBCCS, occurring frequently in the first and second decade of life. Multiple KCOTs can occur in association with other syndromes, such as an orofacial digital syndrome, Ehlers-Danlos syndrome, Simpson Golabi–Behmel syndrome, and Noonan syndrome [1, 6]. The KCOTs seen in NBCCS are multiple, stretching in numbers from 1 to 30, and have a high rate of recurrence. Radiographic features of NBCCS include unilocular or multilocular radiolucent lesions, with smooth or scalloped borders, associated with impacted or displaced teeth and an affinity to grow along the internal aspect of the jaw, resulting in minimal expansion [7]. The radiological differential diagnosis for unilocular or multilocular radiolucency seen in NBCCS includes dentigerous cyst, lateral periodontal cyst, residual cyst, ameloblastoma, simple bone cyst, and odontogenic myxoma [8]. KCOTs associated with NBCCS have a higher potential for recurrence (60%) as compared KCOTs not associated with NBCCS (28%). Recurrence of a KCOT has been documented to occur within two years to 25 years after surgical enucleation [6-7]. Treatment protocol for KCOT includes marsupialization/ enucleation/osseous en bloc resection with supplemental therapies, such as aggressive curettage, cryotherapy, or application of Carnoy solution.

Patients with NBCCS are more susceptible to X-ray radiation hazards; therefore, low-dose imaging modalities should be selected for investigation of NBCCS [1]. Basal cell carcinoma (BCC) is also associated with other syndromes like Bazex syndrome and Torres syndrome; however, when associated with NBCCS, BCC is predominantly seen in adolescent patients and may involve non-sun-exposed areas of the body. The incidence rate of multiple basal cell carcinomas varies widely among different ethnic groups probably because of protective skin pigmentation [6]. Even though multiple BCC was not reported in the present case series, they have been documented in the literature. Calcification of the falx cerebri is one of the most common radiological findings, observed in 37% to 79% of cases associated with NBCCS. Kimonis, et al. found calcification of the falx cerebri to be more frequent among NBCCS patients after 20 years of age [5]. Calcification of the falx cerebri was documented in two out of three patients in the present case series. Rib anomalies are reported in 30% to 60% of patients associated with NBCCS of which bifid ribs are more common as compared to other rib anomalies and present in almost 40% of the NBCCS cases [6]. Two out of three patients in the current case series had bifid ribs. Bifid ribs could be an isolated secondary finding present in the general population but may also be associated with a multisystem disorder like NBCCS and juvenile malignancies like neuroblastomas [9].

Medulloblastoma (now termed primitive neuroectodermal tumor) has been reported in 3% to 5% of NBCCS patients and occurs more frequently within the first two years of life. Histologically, the primitive neuroectodermal tumor is predominantly of the desmoplastic subtype and has a fair prognosis compared to medulloblastoma that occurs in non-syndromic patients [6]. Radiation therapy is not an ideal treatment modality for a primitive neuroectodermal tumor in patients with NBCCS, as they are prone to develop BCC and other intracranial tumors [1]. Patients in the present case series were considerably older than the risk group for the occurrence of a primitive neuroectodermal tumor. Ovarian fibromas and cysts are observed in 25% to 50% of female patients associated with NBCCS. Ovarian fibroma is usually seen in females around 16 to 45 years of age and is detected on pelvic ultrasound [6]. However, the present study does not report any female patient. Once the diagnosis of NBCCS is confirmed, screening must be carried out in other family members to rule out the presence of this hereditary disorder. NBCCS is a hereditary condition with germline mutations in the PTCH1 gene, but 30% to 50% of the cases have also been reported to be sporadic in nature, occurring with new mutations [10]. Future studies could be focused on investigating the PTCH germline mutations with NBCCS.

## Conclusions

We report here rare cases of Gorlin-Goltz syndrome and the importance of a multidisciplinary approach in management. Meticulous extraoral and intraoral examinations, along with radiographs, help in confirming the diagnosis of Gorlin-Goltz​ syndrome. This investigation prompts an early diagnosis and management of the disease, which is very important to prevent recurrence and better survival rates of the concerned patients.

## References

[1] Gupta SR, Jaetli V, Mohanty S, Sharma R, Gupta A (2012). Nevoid basal cell carcinoma syndrome in Indian patients: a clinical and radiological study of 6 cases and review of literature. Oral Surg Oral Med Oral Pathol Oral Radiol.

[2] Gorlin RJ, Goltz RW (1960). Multiple nevoid basal-cell epithelioma, jaw cysts and bifid rib. A syndrome. N Engl J Med.

[3] García de Marcos JA, Dean-Ferrer A, Arroyo Rodríguez S, Calderón-Polanco J, Alamillos Granados FJ, Poblet E (2009 ). Basal cell nevus syndrome: clinical and genetic diagnosis. Oral Maxillofac Surg.

[4] Evans DG, Ladusans EJ, Rimmer S, Burnell LD, Thakker N, Farndon PA (1993). Complications of the naevoid basal cell carcinoma syndrome: results of a population based study. J Med Genet.

[5] Kimonis VE, Mehta SG, Digiovanna JJ, Bale SJ, Pastakia B (2004). Radiological features in 82 patients with nevoid basal cell carcinoma (NBCC or Gorlin) syndrome. Genet Med.

[6] Lo Muzio L (2008). Nevoid basal cell carcinoma syndrome (Gorlin syndrome). Orphanet J Rare Dis.

[7] MacDonald-Jankowski DS (2011). Keratocystic odontogenic tumour: systematic review. Dentomaxillofac Radiol.

[8] Yonetsu K, Bianchi JG, Troulis MJ, Curtin HD (2001). Unusual CT appearance in an odontogenic keratocyst of the mandible: case report. AJNR Am J Neuroradiol.

[9] Schumacher R, Mai A, Gutjahr P (1992). Association of rib anomalies and malignancy in childhood. Eur J Pediatr.

[10] Ortega García de Amezaga A, García Arregui O, Zepeda Nuño S, Acha Sagredo A, Aguirre Urizar JM (2008). Gorlin-Goltz syndrome: clinicopathologic aspects. Med Oral Patol Oral Cir Bucal.

